# Plasma Cystine as a Marker of Acute Stroke Severity

**DOI:** 10.3390/diagnostics15202662

**Published:** 2025-10-21

**Authors:** Alexander Vladimirovich Ivanov, Mikhail Aleksandrovich Popov, Polina Alexandrovna Pudova, Ruslan Andreevich Maslennikov, Valery Vasil’evich Aleksandrin, Maria Pavlovna Galdobina, Maria Petrovna Kruglova, Ekaterina Vladimirovna Silina, Victor Alexandrovich Stupin, Marina Yurievna Maksimova, Aslan Amirkhanovich Kubatiev

**Affiliations:** 1 Institute of General Pathology and Pathophysiology, Baltiyskaya St., 8, 125315 Moscow, Russia; popovcardio88@mail.ru (M.A.P.); polinaklever28@gmail.com (P.A.P.); aleksandrin-54@mail.ru (V.V.A.); masha-5-5@mail.ru (M.P.G.); niiopp@mail.ru (A.A.K.); 2 Moscow Regional Research and Clinical Institute n.a. M.F. Vladimirskiy, Shchepkin St., 61/2, 129110 Moscow, Russia; rusmaslennikov@mail.ru; 3 Department of Pathological Physiology, I.M. Sechenov First Moscow State Medical University (Sechenov University), Trubetskaya St., 8, 119991 Moscow, Russia; marykruglova@live.ru (M.P.K.); silinaekaterina@mail.ru (E.V.S.); 4 Department of Hospital Surgery No. 1, Pirogov Russian National Research Medical University, Ostrovityanova St., 1, 117997 Moscow, Russia; 5 Research Center of Neurology, Volokolamskoye Shosse, 80, 125367 Moscow, Russia; ncnmaximova@mail.ru

**Keywords:** aminothiols, cysteine, cysteine, neurological deficit, stroke markers

## Abstract

**Background/Objectives**: The amino acid cysteine (Cys) plays an important role in the neuronal injury process in stroke. Cys is present in blood plasma in various forms. The relationship between Cys and its forms and the severity of acute stroke has not been sufficiently studied. We investigated the levels of total Cys and two of its forms (reduced Cys and its disulfide (cystine, CysS)) in blood plasma and their influence on stroke severity in patients at admission. **Methods**: A total of 210 patients (39–59 years old) with ischemic stroke and intracerebral or subarachnoid hemorrhage were examined. The contents of the different forms of Cys were determined in the first 10–72 h. Stroke severity was estimated using the National Institutes of Health Stroke Scale (NIHSS) and the modified Rankin Scale (mRs). **Results**: CysS levels < 54 μM were associated with severe (NIHSS > 13) neurological deficit (ischemic stroke: RR = 5.58 and *p* = 0.0021; hemorrhagic stroke: RR = 3.56 and *p* = 0.0003). Smoking and high levels of total Cys and other thiols (glutathione and homocysteine) appear to be factors determining this relationship. **Conclusions**: Low CysS levels may serve as a potential biomarker of acute stroke severity.

## 1. Introduction

Stroke remains a major medical and social problem due to its high prevalence and severe health consequences. According to the Global Burden of Disease expert estimates, trends in morbidity, mortality, and disability-adjusted life years (DALYs) because of stroke have shown a steady decline in recent decades [1]. The incidence of different types of strokes varies widely. Among them, ischemic stroke (IS) comprises 75–80% and hemorrhages (including subarachnoid) comprise up to 20–25%. According to the international TOAST criteria (Trial of Org 10172 in Acute Stroke Treatment), there are IS associated with the pathology of extra- and intracranial arteries (atherothrombotic stroke), cardiogenic embolic stroke, stroke caused by the pathology of small arteries (lacunar stroke), stroke caused by another cause, and stroke of an unknown etiology or caused by two or more possible causes (cryptogenic stroke) [1,2]. Hemorrhagic stroke (HS) is further divided into intracerebral and subarachnoid hemorrhage. It has been proven that the pathogenesis of stroke and the gradual death of neurons are attributed to the universal processes of excitotoxicity, oxidative stress (OS), neuroinflammation, and apoptosis [3].

The most significant risk factors for stroke include arterial hypertension, atherosclerotic stenosis of the internal carotid artery, heart disease (including atrial fibrillation), diabetes mellitus (DM), lipid metabolism disorders, smoking, and excess body weight [2]. The identification of metabolic factors that increase the risk of stroke helps to improve our understanding of its causes.

A closely related metabolite system that deserves increasing attention is that of aminothiols (LMWTs) (cysteine (Cys), homocysteine (Hcy), and glutathione (GSH)). An elevated level of Hcy in blood plasma, or hyperhomocysteinemia (HHcy), is a prothrombogenic and atherogenic factor and a marker of stroke risk [4,5,6]. Numerous studies have found a significant correlation between HHcy and stroke severity/adverse outcome [7,8,9]. In contrast, GSH, the major intracellular thiol and main antioxidant in the cytosol, plays a protective role in stroke [10,11,12].

Cys is a non-essential proteinogenic (unlike Hcy) amino acid obtained mainly from food and partly from methionine. Cys is a rate-limiting substrate for GSH synthesis [13,14]. However, its role is controversial in the Cu-induced oxidation of low-density lipoprotein (LDL), a key factor in the development of atherosclerosis [15,16]. Under ischemic conditions, Cys becomes an important source of H_2_S synthesis at neurotoxic concentrations [17,18,19], as well as an excitotoxic factor that activates NMDA receptors and promotes the release of glutamate from cells [20]. Experimental studies on neuronal cultures have shown that Cys can be neurotoxic at very high concentrations (~1 mM) [21], especially under conditions of oxygen and glucose deprivation (OGD) [22]. However, Cys can also exhibit neurotoxic effects associated with copper overload even at low concentrations (≤1 μM) [23,24]. Thus, Cys can be involved in both neuroprotective and damaging mechanisms, and its role in stroke pathogenesis is unclear.

Unlike GSH, Cys is mainly located in the extracellular environment, partly forming disulfide bonds with proteins or LMWTs. Thus, in blood plasma, the average level of total Cys content (tCys) significantly varies (~120~350 μM), while the concentration of cysteine disulfide (cystine, CysS) ranges from 30 to 60 μM, and the level of the native (reduced) form ranges from ~5 to 15 μM [25,26,27,28]. Despite the relatively high levels of tCys and its fractions in blood plasma, few clinical studies have investigated its association with the risk, severity, and prognosis of stroke. Dose-dependent Cys administration increased the cerebral infarction volume in a rat model of acute IS [29], and a higher Cys intake level negatively impacted stroke risk in women [30]. However, a diet depleted in Cys and methionine led to a decrease in GSH levels in rat brains, leading to an increase in the rats’ sensitivity to OS under hypoxia [13]. The association of tCys with traditional risk factors for stroke (smoking, hypertension, DM, HHcy, obesity, aortic stiffness, and coronary heart disease) has been identified [31,32,33,34,35]. Studies have reported an increase or, conversely, a decrease in this indicator in patients with stroke [36,37], while other studies found no significant effect of plasma tCys levels on the incidence/risk or severity of stroke [4,38,39].

tCys is the sum of all its fractions, which have different activities and different mechanisms of entry into cells. Unlike other Cys GSH fractions, CysS has a high ability to penetrate the blood–brain barrier (BBB) [40]. This suggests that CysS levels may significantly impact the resistance of nervous tissue to ischemia. However, according to our data, there are very few clinical studies of CysS in stroke; in particular, we found only one small study in this area in which high CysS levels were associated with poor stroke outcomes [29]. Another study found no association between CysS and neurological deficit in patients with acute stroke [38].

This study aimed to investigate the associative relationships of the levels of tCys and its fractions (CysS and native or reduced Cys (rCys)) with the severity of acute ischemic/hemorrhagic stroke (measured with the National Institutes of Health Stroke Scale (NIHSS)), as well as functional outcomes after stroke (measured with the modified Ranking Scale (mRs)). Additionally, we aimed to identify factors that could significantly influence these associations.

## 2. Materials and Methods

### 2.1. Patients

This study was conducted from 1 November 2023 to 31 May 2024, following the ethical principles of the Declaration of Helsinki, as outlined by the World Medical Association (1964 and 2004), and with the written, voluntary informed consent of all participants. The study protocol was developed per the CONSORT 2010 recommendations and was approved by the Ethics Committee of the Moscow Regional Research Clinical Institute (protocol No. 7, from 13 July 2023).

This study included a final sample of 210 patients with stroke (aged 39–59) who were admitted to the neurology department of the Moscow Regional Research Clinical Institute within the first 10–72 h after the onset of the neurological disorder symptoms. The subtype of stroke was determined according to the TOAST (Trial of ORG 10172 in Acute Stroke Treatment) classification criteria. The patients had no history of previous cerebrovascular events, such as cerebral infarct, cerebral hemorrhage, or transient ischemic attack. Information on hypertension, type 2 diabetes mellitus, and heart disease (i.e., coronary heart disease, myocardial infarction, valvular disease, and atrial fibrillation) was obtained from their medical histories and clinical data.

The criteria for patient inclusion in this study were as follows:

- Men and women aged up to 60 years inclusive;
- A time from the onset of stroke symptoms to the inclusion in this study of no more than 72 h;
- Patients who suffered their first stroke, verified using magnetic resonance imaging (MRI)/computer tomography (CT) scan of the brain;
- The patient or a disinterested witness signing and dating an informed consent form (if the patient was unable to sign due to physical limitations).

The criteria for non-inclusion of patients from this study were as follows:

- A time from the onset of acute stroke symptoms to the inclusion in this study of more than 72 h;
- Patients with contraindications to CT/MRI (installed pacemaker/neurostimulator/pacemaker; inner ear prosthesis, ferromagnetic or electronic middle ear implants, hemostatic clips, prosthetic heart valves, and any other metal-containing structures or ferromagnetic fragments; and insulin pumps) or inability to undergo the CT/MRI procedure (pronounced claustrophobia, etc.);
- The presence of any neuroimaging (CT/MRI) signs of a brain tumor, arteriovenous malformation, brain abscess, cerebral vascular aneurysm, or edema of the infarct zone, leading to the dislocation of brain structures (malignant course of cerebral infarction);
- Repeated ischemic stroke, hemorrhagic stroke, or a history of unspecified stroke;
- Traumatic brain injury within the past 6 months before screening;
- Patients with a history of surgical intervention on the brain or spinal cord;
- Patients with a history of epilepsy or severe cognitive impairment.

The criteria for the exclusion of patients from this study were as follows:

- Positive blood tests for HIV, syphilis, or hepatitis B and/or C detected at the start of this study;
- The appearance of any diseases or conditions during this study that worsened the patient’s prognosis and made it impossible for the patient to continue participating in the clinical trial;
- Violation of the study protocol, such as incorrect inclusion of patients who did not meet the inclusion criteria, use of prohibited therapy, or other significant protocol violations according to the investigator’s opinion;
- A patient’s refusal to continue participating in this study.

The severity of the neurological disorders was assessed using the NIHSS, and the degree of disability and functional independence was assessed using the mRs [41,42].

All patients underwent a brain MRI using the Magnetom Verio (Siemens, Erlangen, Germany) and Magnetom Symphony (Siemens) devices, with magnetic induction values of 3, 1.5, and 1.5 T, respectively. MRI angiography was performed in 3D-TOF mode to detect intracranial arterial pathology. Brain infarction was identified as a focus of increased MRI signal intensity in the T2, T2 d-f, and diffusion-weighted imaging (DWI) modes, with a decreased diffusion coefficient on the apparent diffusion coefficient (ADC) map.

### 2.2. Laboratory Studies

Venous blood was collected from the patients into 3 mL K3EDTA tubes (Lab-Vac, Heze, China). A volume of 0.35 mL of 0.5 M citrate Na (pH 4.3) was immediately added to the blood. After mixing, the samples were cooled at 4 °C for 3–4 h. The plasma was obtained via blood centrifugation at 350 g for 10 min at room temperature.

Blood samples for the study of hemostasis parameters were collected via venipuncture from the ulnar veins (~12 mL) using disposable vacuum tubes containing 2.5% sodium citrate. The blood was collected into a test tube containing a coagulation activator to analyze the biochemical parameters. The samples were processed no later than 30 min after collection. An ACL TOP 700 hemostasis analyzer (IL Werfen, Barcelona, Spain), an AU 680 biochemical analyzer (Beckman Coulter, Brea, CA, USA), and a PENTRA 120 hematology analyzer (Horiba ABX, Montpellier, France) were used.

The atherogenic coefficient was calculated using the following formula: (total cholesterol—high-density lipoprotein cholesterol (HDL-C))/low-density lipoprotein cholesterol (LDL-C) [43]. The normal value of this indicator for women under the age of 40 is less than 2.5; it can be up to 3.5 at an older age. The normal levels of this indicator for men under and over the age of 40 are less than 2.4 and up to 3.5, respectively.

The determination of total LMWTs in plasma (tCys, tCG, tGSH, and tHcy) was carried out using liquid chromatography with ultraviolet detection, as described in [44]. CysS and rCys levels in plasma were determined using capillary electrophoresis, as described in [28].

### 2.3. Data Processing

Data collection and primary processing (identification and integration of the chromatographic peaks) were performed using MassLynx v4.1 (Waters, Milford, MA, USA) and the Elforun software v. 4.2.5 (Lumex, St. Petersburg, Russia). Statistical data analysis was performed using SPSS Statistics v. 22 (IBM, Armonk, NY, USA). Quantitative indicators are expressed as medians (along with the 1st and 3rd quartiles). The optimal cut-off values for the variables were determined using receiver operating characteristic (ROC) analysis. Logistic regression analysis was performed to assess the impact of the variables on the NIHSS and mRs scores. A cut-off value of NIHSS ≥ 14 or, in some cases, NIHSS > 10 was used to distinguish severe and mild strokes. We used a cut-off value of mRs ≤ 3 to divide the cohort according to the functional state. Binomial indicators (variable analysis) were compared based on the relative risk ratio (RR) and odds ratio (OR); *p* < 0.05 indicated a significant difference. The Mann–Whitney test was used to compare the variables between groups. Post hoc analysis was additionally performed to assess the completeness of the sample size (α = 0.05). Correlation analysis was performed using the Spearman method. A two-sided critical significance level (*p*) was used for all comparisons and tests. In the case of multiple comparisons, the Holm–Bonferroni method was used to correct the *p*-value.

## 3. Results

The demographic characteristics and laboratory data of the patients are presented in Table 1. The levels of total LMWTs, CysS, and rCys could be determined in 203 (96.7%), 202 (96.2%), and 186 (88.6%) patients, respectively. Most patients were men. This patient cohort displayed a high frequency of smoking, overweight, dyslipidemia, and arterial hypertension. About half of the patients reported regular consumption of alcoholic beverages.

According to the Spearman correlation analysis, none of the LMWT parameters had a statistically significant association with stroke severity, as assessed using the NIHSS. The total LMWT levels were positively associated with each other, except for tGSH and tCys (Table 2). CysS and rCys levels were not associated with each other but showed statistically significant and multidirectional associations with tHcy and tGSH. Only the associations of tGSH level with cholesterol and tCys were found when comparing LMWT levels with other clinical and laboratory parameters.

When dividing the patient cohort into two groups (IS and HS), we did not find statistically significant differences between them in the levels of tCys and its fractions (CysS and rCys), as well as in other studied parameters, except for tGSH. The tGSH level in the IS group was higher (see Table 1; *p* = 0.01). In addition, the total score, as assessed using the NIHSS, was lower in the IS group compared with the HS group (*p* = 0.019).

When dividing the patient cohort into two groups according to the criterion of mRs 0–3 and mRs > 3, we did not find statistically significant differences regarding any indicators. The univariate logistic analysis did not reveal a reliable predictor among all the LMWTs studied.

CysS was the only parameter among all LMWTs that demonstrated significant differences when dividing the patient cohort into two groups based on the criterion of NIHSS ≤ 13 (Table 3). As shown in the table, the CysS level was higher in the group of patients with NIHSS ≤ 13 than in the group of patients with severe stroke. The univariate logistic analysis also showed that, in this case, CysS was the only reliable predictor among all LMWTs (OR = 1.036; 95% CI: 1.012–1.06; *p* = 0.0033).

The optimal cut-off point for CysS to distinguish between the two groups was determined to be 54 μM based on the ROC analysis (AUC: 0.64, 95% CI: 0.549–0.73, *p* = 0.0063, sensitivity: 0.448, and specificity: 0.837; see Supplemental Materials, Figure S1). Using this cut-off point, it was found that patients with high CysS levels (>54 μM) were characterized by a significantly lower risk of severe neurological deficits (NIHSS > 13) compared with patients with CysS levels ≤ 54 μM (Table 4). This was true for both the IS and HS subgroups; however, post hoc analysis showed that the sample size was insufficient for a separate study of these subgroups, especially for HS.

No significant effect of stroke risk factors (atrial fibrillation, hypertension, DM2, dyslipidemia, high atherogenic coefficient, CAD, smoking, alcohol drinking, and HHcy) on CysS levels was found based on a multivariate analysis of variance. Dividing the cohort by these stroke risk factors also revealed no significant differences in CysS levels between the respective patient groups using the Mann–Whitney method (Supplemental Materials, Table S1).

Since LMWTs showed a fairly strong association with each other, we investigated the effect of their levels on the association between CysS and the risk of severe neurological deficit. To achieve this, we first divided the patient cohort into three groups (tertiles) according to the levels of some analytes and then calculated the risk of NIHSS > 13 at a CysS cut-off of 54 μM in the first and third tertiles, as shown in Table 5. For patients with high levels of thiols (tCys, tGSH, and tHcy, but not tCG), the CysS level’s association with stroke severity was more pronounced and reliable than in patients with low levels. When dividing the cohort into tertiles based on rCys, we observed an association between the CysS level and the risk of NIHSS > 13, which was present at both low and high rCys levels. However, the rather low power of the post hoc analysis indicates that a significantly larger sample size is required to confidently detect the effect of thiols on the association of CysS with stroke severity.

**Figure 1:**
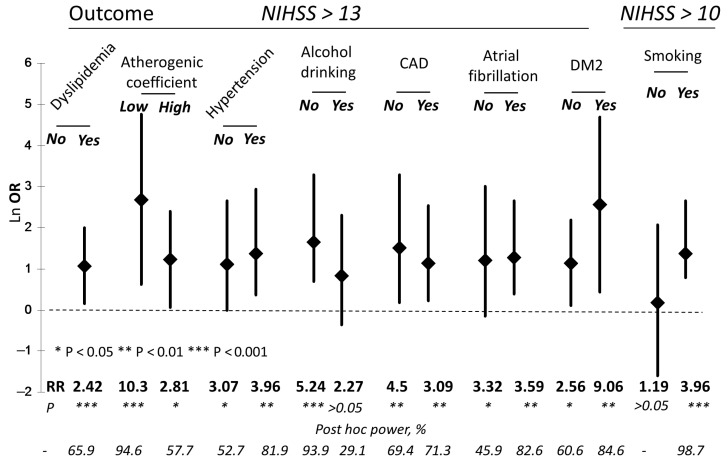
The influence of stroke risk factors on the association of low CysS levels (<54 μM) with neurological deficit. CAD, coronary artery disease; DM2, type 2 diabetes mellitus; Ln OR, odds ratio natural logarithm; RR, relative risk ratio; NIHSS, National Institutes of Health Stroke Scale.

The influence of various stroke risk factors on the association between CysS and neurological deficit was also investigated. Among the 21 patients without dyslipidemia and with a CysS level > 54 μM, we did not observe any patients with NIHSS > 9, while among the 38 patients with a CysS level ≤ 54 μM and without dyslipidemia, the incidence of NIHSS > 9 was 42.1% (*p* = 10^−4^). Simultaneously, a low CysS level (≤54 μM) was not linked to an increased risk of NIHSS > 9 in patients with dyslipidemia (RR = 1.28; *p* > 0.05). However, when the cut-off point for the NIHSS was increased to 13, we observed a higher risk of severe stroke in patients with dyslipidemia who had a CysS level ≤ 54 μM (Figure 1); however, the sample size was insufficient to draw a definite conclusion.

A strong association between low CysS levels and an increased risk of NIHSS > 13 was observed in patients with an atherogenic coefficient in the normal range (Figure 1). The same association was also observed in patients with an elevated atherogenic coefficient, but with significantly lower reliability. We found no significant changes in the association of CysS with NIHSS level when dividing the cohort by binary risk factors such as hypertension, DM2, CAD, and atrial fibrillation (Figure 1).

A statistically significant association between CysS levels and the risk of NIHSS > 13 was found in the non-drinking group but not in the alcohol-consuming group (Figure 1). Similarly, the effect of smoking was also investigated. However, since there were no patients with NIHSS > 13 in the non-smoking group with CysS > 54 μM, the cut-off point for neurological deficit was lowered to NIHSS > 10. Nevertheless, a significant association between a low CysS level and the risk of NIHSS > 10 was only found among smoking patients, as shown in Figure 1.

## 4. Discussion

In this study, we found an association of low (<54 μM) plasma CysS levels with the development of severe neurological deficit in patients with ischemic and hemorrhagic stroke, with this marker demonstrating fairly high specificity. However, its sensitivity was insufficient for triage or prognostic use in the acute period of stroke because a significant proportion of patients with CysS levels of less than 54 μM were characterized by moderate neurological deficit. This association was observed both in the presence and absence of stroke risk factors, such as dyslipidemia, arterial hypertension, coronary heart disease, and atrial fibrillation. This association is likely more characteristic of patients with high levels of tCys, tHcy, or tGSH, but larger-scale studies are required to test this hypothesis. In addition, our results indicate that smoking probably plays a significant role in the association of low CysS levels with the risk of severe stroke. Thus, our data indicate that CysS may function as a protective factor or risk marker in acute stroke. These results are not consistent with an earlier study [29], which found no association between CysS levels and NIHSS scores on the first day of stroke. The most obvious reason for this difference is the small sample size (36 patients).

**Figure 2:**
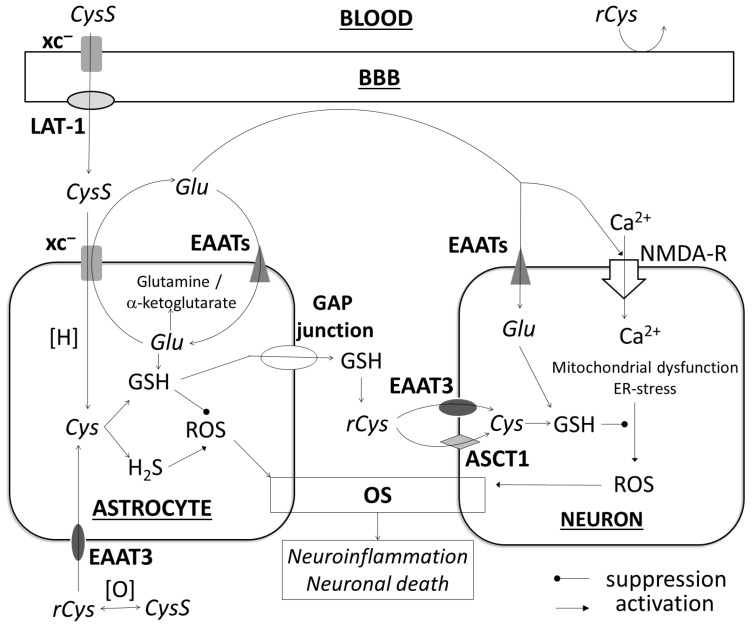
The role of cysteine transport in brain damage and glutamate excitotoxicity. ASCT1, neutral amino acid transporter A; BBB, blood–brain barrier; Cys, cysteine; CysS, cysteine; EAATs, excitatory amino acid transporters; EAAT3, excitatory amino acid transporter 3; ER, endoplasmic reticulum; Glu, glutamate; GSH, glutathione; LAT-1, large neutral amino acid transporter 1; NMDA-R, N-methyl-D-aspartate receptor; OS, oxidative stress; rCys, reduced cysteine; ROS, reactive oxygen species; xc^−^, cystine/glutamate transporter; [O], oxidation.

CysS can be considered a part of the extracellular Cys pool as well as a bioavailable form of Cys for the brain [40]. However, the mechanisms for supplying neurons with Cys are complex and involve the coordinated interaction of several transport systems of various cells, primarily astrocytes and neurons. Cys can enter cells in the form of rCys or CysS. The transfer of rCys into cells is mainly carried out by the excitatory amino acid transporter 3 (EAAT3) in humans or the excitatory amino acid carrier 1 (EAAC1, an analog of EAAT3) in rodents [45], as shown in Figure 2. The EAAT family is also involved in the transport of many amino acids, including glutamate. The endocytosis of CysS occurs via the xc^−^ transporter, but in this case, it is exchanged for glutamic acid in a 1:1 ratio [46]. In rats, xc^−^ is expressed in the brain in astrocytes, while in humans and mice, it is additionally expressed in neurons [47]. CysS is captured by the endothelium from the plasma via xc^−^ and is transferred to the intercellular space via the large neutral amino acid transporter 1 (LAT-1) [40]. Thus, the bioavailability of Cys is generally determined by the rCys, CysS, and GSH transport systems (Figure 2). Having analyzed the role of Cys in neuronal damage, its effect is clearly determined by the balance of its intracellular metabolism and transport mechanisms, which are closely related to glutamate, as schematically shown in Figure 2. xc^−^ and EAAT3/EAAC1 form a dynamic system of processes involving glutamate uptake and release by astrocytes, in which an increase in CysS uptake must be compensated by an increase in glutamate uptake to prevent the activation of NMDA receptors in neurons. The GSH and H_2_S synthesis systems also compete for the initial substrate, while H_2_S can have opposite effects depending on its concentration.

While CysS is the main source for astrocytes with a high level of xc^−^ expression, neurons expressing mainly EAAT3/EAAC1 and the neutral amino acid transporter A (ASCT1) use mainly rCys, which is released by gliocytes in the form of GSH via the gap junction and hydrolyzed to cysteine in the intercellular space [48]. In normal mice, the main source of Cys in neurons is rCys, and CysS transport plays a secondary role [49]. In a mouse model deficient in EAAC1 (the human analog of EAAT3), a significant decrease in brain GSH levels and an increased sensitivity to OS were found [40], whereas xc^−^-knockout mice had normal brain GSH levels [50,51]. Genetic deletion of EAAC1 aggravates ischemia-induced neuronal death [48]. Other studies showed that the observed increase in xc^−^ expression in astrocytes (in vivo and in vitro) plays an important role in maintaining the intracellular GSH pool and protecting against neuronal ferroptosis under hypoxia [52,53,54], indicating an increasing neuroprotective role of CysS during ischemia. Conversely, inhibition of xc^−^ expression leads to the depletion of GSH and a decrease in its redox status [53]. The leading role of xc^−^ in the expression of the neuroprotector erythropoietin under hypoxic conditions has also been reported [53].

Simultaneously, xc^−^ participates in neuronal death mechanisms. More than 99% of total glutamate is located in cells, and xc^−^ provides transport for 60–80% of the entire extracellular glutamate pool in the brain, which makes the activation of this transporter a significant factor in nerve tissue damage [45,55]. This is supported by a previous study showing that xc^−^ inhibition suppressed ischemia/reperfusion-induced elevation of extracellular glutamate and NMDA receptor activation, and attenuated ischemia-gated currents and cell death after OGD [56,57]. Thus, it can be assumed that if the suppression of CysS uptake has a generally positive effect, and xc^−^ is considered a therapeutic target in stroke, then the CysS level can act as a marker; its increase in blood plasma may be associated with less neuronal damage and a milder course of stroke.

The key role of xc^−^ astrocytes in IL-1β-induced neuronal damage was also revealed in a mixed culture of cortical neurons and astrocytes [58]. Different results were obtained in different models of ischemic stroke in mice with xc^−^ light-chain knockout. In some cases, there were no changes in cerebral infarction volume and stroke incidence, while in other cases, these indicators were lower in knockout mice than in wild-type animals [52,56]. In a rat model of ischemic stroke, the use of N-acetylcysteine, which can enter cells bypassing EAATs and xc^−^, led to a significant suppression of xc^−^ expression and an increase in glutamate transporter-1 (GLT-1) expression, which captures glutamate, leading to a decrease in the extracellular pool of glutamate in the brain [59]. This indicates the presence of a negative control mechanism for CysS transport from the intracellular pool.

It has been previously established that the Cys content in the brain increases during ischemia due to the increased degradation and decreased synthesis of GSH [60,61]. The latter, given its high intracellular concentration, can also be considered a glutamate depot. Therefore, the inhibition of GSH (γ-glutamylcysteine ligase) synthesis is accompanied by an increase in the concentration of cytosolic glutamate in neurons, whereas its activation leads to a decrease in the concentration of glutamate [62]. In this context, if we consider the increase in tCys or CysS levels as a consequence of the disturbance of GSH metabolism during brain injury, then we would expect an association of tCys and/or its fractions with the volume of cerebral infarction or penumbra. However, the absence of such a result means that the release of Cys from damaged brain tissue does not significantly contribute to the pool of circulating tCys. In addition, the possible increase in tCys levels due to this reason may be compensated by a decrease in the formation of Cys from Hcy due to the mobilization of pyridoxal-5-phosphate (the coenzyme cystathionine beta-synthase (CβS) is necessary for this pathway), which, under conditions of systemic inflammatory reactions, is used primarily for tryptophan catabolism [37].

CβS’s main function is the formation of cystathionine from Hcy, but its H_2_S-producing activity becomes the most pathogenetically significant in stroke, mainly due to glial cells (microglia and astrocytes). Both Hcy and Cys can act as substrates in this process. Experimental stroke research has found that CβS activity plays both neuroprotective and damaging roles. Thus, deficiency of this enzyme in the MCAO mouse model caused additional activation of the inflammatory factor NF-κB, followed by increased expressions of the cytokines IL-1β, IL-6, and TNFα. Administration of NaHS suppressed this effect [63]. MCAO led to the suppression of CβS expression in the brain, which was associated with the conversion of microglia/macrophages into the pro-inflammatory M1 phenotype, while stimulation of CβS expression shifted these cells to the anti-inflammatory phenotype [64,65]. These findings align with the results of another study [66] that used the SAH model to demonstrate the neuroprotective effect of intracerebroventricular administration of Cys, which was associated with an increase in the H_2_S-generating activity of CβS, expressed as a reduction in cerebral edema, improved neurobehavioral function, and attenuated neuronal cell death.

Simultaneously, several studies have demonstrated the negative role of CβS. In a MCAO rat model, inhibition of CβS led to a decrease in the volume of cerebral infarction, whereas Cys administration increased the H_2_S levels in the brain and contributed to an increase in infarction [18,67]. OGD in astrocyte cultures was found to reduce cell survival and was associated with a significant increase in H_2_S levels [65]. H_2_S is a second messenger that affects many enzymes and is believed to cause neurotoxicity by inhibiting monoamine oxidase, cholinesterase, Na+/K+-ATPase, and cytochrome c oxidase. It also enhances the effects of glutamate [17,68]. Finally, according to a study examining cerebrospinal fluid (CSF) samples from patients with SAH, the upregulated expression of CβS was closely associated with the inflammatory response and neurological deficits [69].

Cys appears to play an important and ambiguous role in Cu-mediated mechanisms of OS development and neuronal cell death, as well as atherogenesis. Thus, in blood plasma, Cys and CysS inhibit Cu-mediated LDL oxidation by binding and/or reducing Cu [16]; however, they simultaneously potentiate Hcy’s ability to oxidize LDL in the presence of Cu [16]. Cu is essential for the functioning of vital proteins such as cytochrome c oxidase, superoxide dismutases 1 and 3, tyrosinase, and amino oxidases. Maintaining the GSH/GSSG redox balance in the cell is essential for the normal functioning of the Cu transport system and the maturation of Cu-containing enzymes, as GSH serves as a cytosolic Cu transporter and also regulates the activity of the Cu chaperone Atox1 [70]. GSH oxidation or inhibition of synthesis disrupts Cu transport, leading to its accumulation in cellular compartments. In cerebral ischemia–reperfusion injury, the level of copper ions increases [71]. In contrast, administration of the GSH precursor, N-acetylcysteine, resulted in a decrease in Cu accumulation in the brain [72] and attenuated the OS induced by Cu overload [73], but it did not significantly affect cell cultures’ survival when cuproptosis was induced in them [74]. Meanwhile, it has been shown in vitro that Cys can induce Cu accumulation in cells via an unclear mechanism not associated with GSH synthesis or the formation of Cu-Cys complexes [72]. As a variable-valence metal, Cu catalyzes the formation of ROS, with Cys serving as the primary substrate. For example, it has been shown that the presence of Cys and submicromolar concentrations of Cu resulted in massive hydroxyl radical generation and cell death in cultured primary cortical neurons, while no cytotoxic effects were observed in the presence of Cu alone [23,24]. Additionally, cysteine’s neurotoxic effect can be induced by disrupting mitochondrial respiration via an oxidant-based mechanism, limiting intracellular iron availability [75,76].

CysS is one of the oxidized forms of Cys. High CysS and low reduced GSH levels were associated with a risk of future death in a high-risk population with CAD; moreover, this effect was independent of, and additive to, that of CRP levels [77]. This suggests that CysS acts as a marker of the risk of adverse cardiovascular outcomes in the long term. Nevertheless, our results and the literature data suggest that CysS may be a factor in antioxidant protection, but not a marker of OS in acute stroke, although many of the above-described mechanisms of its action are associated with OS.

This study had several limitations. The insufficient sample size may explain why we did not find a significant association between low CysS levels and an increased risk of severe stroke in patients with low levels of other LMWTs (tCys, tHcy, and tGSH). Since there was a clear correlation between CysS and these thiols, the low frequency of patients with high CysS levels and low levels of other thiols was the major limiting factor, resulting in a significance level outside the significance limits. The insufficient sample size likely limited the ability to detect the impact of factors such as dyslipidemia, atrial fibrillation, and smoking on stroke severity in patients with high CysS levels. Blood samples were taken from patients upon admission, which could also have influenced the variability of the results. Elderly patients were not included in this study. Given that plasma Hcy levels increase with age [78], while GSH levels [79] and aminothiol redox status [80] decrease, it can be assumed that an association of CysS levels with stroke severity would be observed in elderly individuals. The use of several drugs (for example, aspirin and B vitamins) could also have influenced the results. Thus, additional and larger-scale studies are required to address these issues.

The intensive development of non-invasive MRI-based technologies, such as quantitative susceptibility mapping (QSM), allows us to detect the infarction zone as well as obtain a detailed picture of changes in brain tissue associated with the metabolism of iron, myelin, and brain oxygen saturation, making this method valuable in the diagnosis and prognosis of stroke [81,82]. Therefore, the combined use of QSM with aminothiol analysis could be useful for integrating data on circulating thiols with local brain tissue changes in studies of promising approaches to the treatment of acute stroke and identifying pathophysiological determinants of the effectiveness of correction of Cys and GSH metabolism in the brain.

Many inflammatory indicators (amyloid A, CRP, matrix metalloproteinases, myeloperoxidase, phospholipase A2, and others) [83] and some OS indicators (malondialdehyde, protein carbonyls, and NO metabolites) [84] have been identified as risk markers for stroke severity and prognosis. Hence, it is of interest to identify the links between CysS and these markers and the possibility of influencing them via cysteine metabolism. Thus, clinical and experimental studies have demonstrated the ability of ingestion of CysS with theanine (a precursor of glutamic acid for GSH synthesis) to suppress inflammatory reactions and a decrease in tissue GSH during surgical interventions [85].

## 5. Conclusions

In this study, we revealed that low plasma cystine levels may be a potential biomarker of the severity of both ischemic and hemorrhagic stroke for the first time. Smoking and high levels of total thiols (cysteine, glutathione, and homocysteine) appear to be factors determining this relationship. Given the important role of cystine in the excitotoxicity and antioxidant protection mechanisms of nervous tissue, the results obtained indicate the protective potential of cystine in stroke.

## Figures and Tables

**Table 1:** Characteristics of patients with stroke included in this study.

Characteristics	Total	IS	HS
Number of patients	210	138	72
Stroke type and subtype		AT—74 (53.6%) CE—37 (26.8%) Lac—10 (7.2%) Other—17 (12.3%)	ICH—13 (18.1%) SAH—59 (81.9%)
Age, years (Q1; Q3)	55 (50; 57)	55 (52; 57)	55 (49; 57)
Male/female	161/49	103/35	58/14
NIHSS	7.5 (6; 10)	7 (6; 10)	9 (7; 15) *
mRs	3 (2; 3)	3 (2; 3)	3 (2; 3)
Risk factors			
Hypertension, *n* (%)	113 (53.8)	73 (52.9)	40 (55.6)
DM2, *n* (%)	66 (31.4)	40 (29.0)	26 (36.1)
Dyslipidemia, *n* (%)	148 (70.5)	95 (68.8)	53 (73.6)
HHcy: tHcy > 15 μM (%)	44 (21.7)	31 (23.0)	13 (19.1)
CAD, *n* (%)	110 (52.4)	73 (52.9)	37 (51.4)
Atrial fibrillation, *n* (%)	132 (62.9)	91 (65.5)	41 (57.7)
Current cigarette smoking, *n* (%)	179 (85.2)	115 (82.7)	64 (90.1)
Alcohol drinking, *n* (%)	104 (49.5)	66 (47.8)	38 (52.8)
Body mass index	27.6 (27.2; 28.0)	28 (27.2; 28)	27.6 (27.2; 28.6)
Body mass index > 25 kg/m^2^, *n* (%)	198 (94.3)	130 (93.5)	68 (95.8)
Laboratory findings			
Total cholesterol, mM	3.5 (1.7; 4.0)	3.5 (1.7; 4.0)	3.5 (1.7; 3.9)
TGs, mM	2.1 (1.5; 2.7)	2.1 (1.5; 2.7)	2.1 (1.7; 2.7)
HDL-C, mM	1.2 (1.0; 1.4)	1.2 (1.0; 1.4)	1.2 (1.0; 1.3)
LDL-C, mM	2.4 (2.2; 3.4)	2.4 (2.2; 3.2)	2.4 (2.2; 3.4)
High atherogenic coefficient (%)	116 (55.2)	77 (55.4)	39 (54.9)
Glucose, mM	4.9 (4.2; 5.1)	4.9 (4.7; 6.1)	4.8 (4.7; 5.1)
aPTT, s	33 (27; 35)	33 (27; 35)	33 (27; 35)
Fibrinogen, g/L	3.8 (3.7; 3.9)	3.8 (3.6; 4.0)	3.8 (3.7; 3.9)
WBCs, 10^9^/L	7.0 (5.25–8.0)	7.0 (5.0–8.0)	7.0 (6.0–8.0)
PLT, 10^9^/L	278 (234; 312)	289 (234; 312)	278 (234; 311)
CRP, mg/L	4 (3;6)	4 (3; 6)	4 (3; 7)
IL-6, pg/mL	4 (3;6)	4 (3; 6)	4 (3; 6)
Ferritin, μg/L	75 (45; 90)	75 (45; 90)	75 (45; 90)
LMWTs			
tCys, μM	211 (173; 253)	213 (171; 251)	211 (179; 255)
tGSH, μM	2.9 (2.3; 3.8)	3.17 (2.48; 3.89)	2.70 (2.09; 3.34) *
tHcy, μM	11.3 (8.2; 14.7)	11.5 (8.6; 14.8)	10.0 (7.8; 14.4)
tCG, μM	20.0 (16.5; 25.0)	20.0 (16.5; 24.8)	20.1 (16.5; 26.1)
rCys, μM	13.7 (9.7; 19.1)	12.9 (9.9; 19.1)	16.6 (9.3; 18.9)
CysS, μM	49.8 (40.1; 58.3)	49.3 (39.6; 57.4)	51.1 (40.9; 61.1)

* corrected *p* < 0.05. aPTT, activated partial thromboplastin time; AT, atherothrombotic stroke; CE, cardioembolic stroke; DM2, type 2 diabetes mellitus; CAD, coronary artery disease; CRP, C-reactive protein; CysS, cystine; ICH, intracerebral hemorrhage; IL-6, interleukin 6; IS, ischemic stroke; HDL-C, high-density lipoprotein cholesterol; HHcy, hyperhomocysteinemia; HS, hemorrhagic stroke; Lac, lacunar stroke; LDL-C, low-density lipoprotein cholesterol; LMWTs, low-molecular-weight aminothiols; mRs, modified Rankin Scale; NIHSS, National Institutes of Health Stroke Scale; PLT, platelets; rCys, reduced cysteine; SAH, subarachnoid hemorrhage; tCG, total cysteinylglycine; tCys, total cysteine; TGs, triglycerides; tGSH, total glutathione; tHcy, total homocysteine; WBCs, white blood cells.

**Table 2:** Spearman correlation of LMWTs in the whole cohort.

LMWTs	tCys	CysS	rCys	tCG	tHcy	tGSH
tCys	-	0.393 ***	−0.178	0.551 ***	0.645 ***	0.160
CysS		-	−0.041	0.041	0.260 **	0.229 *
rCys			-	−0.149	−0.360 ***	−0.230 *
tCG				-	0.450 ***	0.221 *
tHcy					-	0.371 ***
Cholesterol	0.173	−0.162	0.077	−0.027	−0.193	−0.208 *

* *p* < 0.05, ** *p* < 0.01, and *** *p* < 0.001. CysS, cysteine; LMWTs, low-molecular-weight aminothiols; rCys, reduced cysteine; tCG, total cysteinylglycine; tCys, total cysteine; tGSH, total glutathione; tHcy, total homocysteine.

**Table 3:** Comparison of LMWT levels in patients with different neurological deficits.

Variable	NIHSS ≤ 13 (N = 168)	NIHSS > 13 (N = 42)	P^Mann-U^
Age, years	55 (50.3; 57)	55 (49; 58)	0.72
tCys, μM	216 (172; 254)	204 (173; 252)	0.604
tGSH, μM	2.89 (2.23; 3.85)	3.11 (2.60; 3.52)	0.394
tCG, μM	19.1 (16.4; 24.8)	21.1 (18.3; 28.1)	0.088
tHcy, μM	11.3 (8.0; 14.8)	11.1 (9.0; 14.2)	0.872
CysS, μM	51.1 (41.0; 59.4)	44.7 (34.5; 53.4)	0.0063 *
rCys, μM	13.7 (8.9; 18.8)	13.3 (10.0; 20.3)	0.6

* Post hoc power of 92.3%. CysS, cysteine; LMWTs, low-molecular-weight aminothiols; NIHSS, National Institutes of Health Stroke Scale; rCys, reduced cysteine; tCG, total cysteinylglycine; tCys, total cysteine; tGSH, total glutathione; tHcy, total homocysteine.

**Table 4:** Association of CysS level with risk of severe neurological deficit.

CysS, μM	Proportion of Patients with NIHSS > 13 (%)	RR (*p*)	OR (95% CI)	Post Hoc Power, %
Whole cohort				
≤54	34 out of 124 (27.4)	3.56 (0.0003)	4.53 (1.80–11.39)	95.3
>54	6 out of 78 (7.7)			
IS				
≤54	20 out of 87 (23)	5.29 (0.003)	6.57 (1.46–29.51)	84.2
>54	2 out of 46 (4.3)			
HS				
≤54	14 out of 37 (37.8)	3.12 (0.0069)	4.41 (1.28–15.23)	70.2
>54	4 out of 33 (12.1)			

CysS, cysteine; NIHSS, National Institutes of Health Stroke Scale; IS, ischemic stroke; HS, hemorrhagic stroke; OR, odds ratio; RR, relative risk ratio.

**Table 5:** The influence of LMWTs on the association of CysS with the risk of severe neurological deficit.

Factor	N^NIHSS>13^/ N^CysS≤54 μM^	N^NIHSS>13^/ N^CysS>54 μM^	RR (*p*)	OR (95% CI)	Post Hoc Power, %
T1 tGSH (0.64–2.48 μM)	7/43	2/23	1.87 (>0.05)	2.042 (0.39–10.75)	N/d
T3 tGSH (3.42–22.3 μM)	10/34	2/30	4.41 (0.01)	5.83 (1.16–29.27)	65.2
T1 tHcy (3.1–9.1 μM)	9/50	1/16	2.88 (>0.05)	3.29 (0.38–28.24)	N/d
T3 tHcy (13.3–30.9 μM)	9/30	2/34	5.1 (0.005)	6.86 (1.35–34.93)	72.6
T1 tCys (62.4–185 μM)	15/54	1/12	3.33 (>0.05)	4.23 (0.50–35.67)	N/d
T3 tCys (239–385 μM)	11/30	2/34	6.23 (0.0013)	9.26 (1.85–46.34)	87.3
T1 tCG (9.1–17.0 μM)	6/38	3/28	1.47 (>0.05)	1.56 (0.36–6.87)	N/d
T3 tCG (23.6–73.7 μM)	9/44	5/20	0.82 (>0.05)	0.77 (0.22–2.69)	N/d
T1 rCys (0.87–10.6 μM)	10/34	3/28	2.75 (0.036)	3.47 (0.85–14.2)	42
T3 rCys (17.3–51.6 μM)	14/40	2/22	3.85 (0.013)	5.39 (1.1–26.46)	62.6

CysS, cysteine; LMWTs, low-molecular-weight aminothiols; N, number of patients; NIHSS, National Institutes of Health Stroke Scale; OR, odds ratio; rCys, reduced cysteine; RR, relative risk ratio; T1, first tertile; T3, third tertile; tCG, total cysteinylglycine; tCys, total cysteine; tGSH, total glutathione; tHcy, total homocysteine.

## Data Availability

The complete raw data are not publicly available due to local data protection laws, but de-identified data can be made available upon reasonable request from the corresponding author.

## Supplementary Materials

The following supporting information can be downloaded at: https://www.mdpi.com/article/10.3390/diagnostics15202662/s1, Figure S1: ROC-curve for plasma CysS in stroke patients; Table S1: The influence of stroke risk factors on CysS levels (in µM).

## Abbreviations

The following abbreviations were used in this manuscript:

ADC	Apparent diffusion coefficient
aPTT	Activated partial thromboplastin time
ASCT1	Neutral amino acid transporter A
AT	Atherothrombotic stroke
BBB	Blood–brain barrier
CAD	Coronary artery disease
CE	Cardioembolic stroke
CG	Cysteinylglycine
CRP	C-reactive protein
CSF	Cerebrospinal fluid
CT	Computer tomography
Cys	Cysteine
CysS	Cystine
CβS	Cystathionine beta-synthase
DM2	Type 2 diabetes mellitus
DWI	Diffusion-weighted imaging
EAAT	Excitatory amino acid transporter
ER	Endoplasmic reticulum
EAAC1	Excitatory amino acid carrier 1
GLT-1	Glutamate transporter-1
Glu	Glutamate
GSH	Glutathione
Hcy	Homocysteine
HDL-C	High-density lipoprotein cholesterol
HHcy	Hyperhomocysteinemia
HS	Hemorrhagic stroke
ICH	Intracerebral hemorrhage
IL-1β	Interleukin 1β
IL-6	Interleukin 6
IS	Ischemic stroke
Lac	Lacunar stroke
LAT-1	Large neutral amino acid transporter 1
LDL-C	Low-density lipoprotein cholesterol
LMWTs	Low-molecular-weight aminothiols
MCAO	Middle cerebral artery occlusion
MRI	Magnetic resonance imaging
mRs	Modified Rankin Scale
NF-κB	Nuclear factor kappa b
NIHSS	National Institutes of Health Stroke Scale
NMDA	N-methyl-D-aspartate
OGD	Oxygen and glucose deprivation
OR	Odds ratio
OS	Oxidative stress
PLT	Platelets
QSM	Quantitative susceptibility mapping
rCys	Reduced cysteine
ROC	Receiver operating characteristic
ROS	Reactive oxygen species
RR	Relative risk ratio
SAH	Subarachnoid hemorrhage
T1	First tertile
T3	Third tertile
tCG	Total cysteinylglycine
tCys	Total cysteine
TGs	Triglycerides
tGSH	Total glutathione
tHcy	Total homocysteine
TNFα	Tumor necrosis factor-alpha
TOAST	Trial of ORG 10172 in Acute Stroke Treatment
WBCs	White blood cells
xc^−^	Cystine/glutamate transporter
