# Supplementary material for: Plasma Cystine as a Marker of Acute Stroke Severity
Source: Diagnostics (Basel). 2025 Oct 21;15(20):2662. doi: 10.3390/diagnostics15202662 (PMC12564503; doi:10.3390/diagnostics15202662)
Supplement: Supplementary file 1 [file diagnostics-15-02662-s001.zip › diagnostics-3911926-supplementary.pdf]

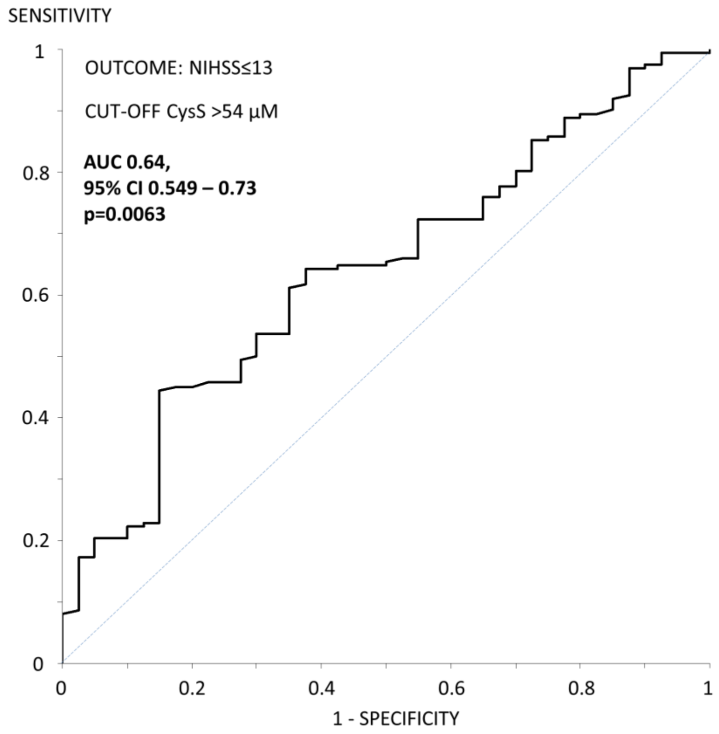

Figure S1. ROC-curve for plasma CysS in stroke patients.

Table S1. The influence of stroke risk factors on CysS levels (in µM).

Risk factor	Absent	Present	$P^{Mann\text{-}U}$
DM2	49.4 (40.1; 57.6)	52.3 (39.3; 59.6)	0.785
Atrial fibrillation	50.0 (32.5; 57.0)	49.8 (41.9; 60.2)	0.164
CAD	48.5 (39.3; 57.8)	50.2 (40.6; 58.5)	0.421
Current cigarette smoking	48.0 (35.7; 57.8)	50.0 (40.7; 58.7)	0.266
High atherogenic coefficient	50.2 (41.9; 56.3)	49.3 (40.0; 60.3)	0.884
Alcohol drinking	50.2 (38.4; 58.0)	49.5 (40.2; 63.6)	0.592
Hypertension	50.0 (41.1; 58.7)	49.7 (39.5; 58.3)	0.573
Dyslipidemia	49.7 (37.9; 59.1)	50.0 (40.2; 58.4)	0.851
HHcy	48.8 (39.1; 57.7)	54.2 (44.9; 64.0)	0.387*

* Holm–Bonferroni corrected

DM2, type 2 diabetes mellitus; CAD, coronary artery disease; CysS, cystine; HHcy, hyperhomocysteinemia.
